# Differences in medical specialist utilization among older people in need of long-term care – results from German health claims data

**DOI:** 10.1186/s12939-020-1130-z

**Published:** 2020-02-07

**Authors:** Maike Schulz, Jonas Czwikla, Chrysanthi Tsiasioti, Antje Schwinger, Daniel Gand, Guido Schmiemann, Annika Schmidt, Karin Wolf-Ostermann, Stephan Kloep, Franziska Heinze, Heinz Rothgang

**Affiliations:** 1 University of Bremen, SOCIUM Research Center on Inequality and Social Policy, Mary-Somerville-Straße 5, 28359 Bremen, Germany; 2 University of Bremen, Institute for Public Health and Nursing Research (IPP), Grazer Straße 4, 28359 Bremen, Germany; 3 Research Institute of the Local Health Care Funds (WIdO), P.O. Box 11 02 46, 10832 Berlin, Germany; 4 University of Bremen, Competence Center for Clinical Trials (KKSB), Linzer Straße 4, 28359 Bremen, Germany; 5 University of Bremen, High-Profile Area Health Sciences, Bremen, Germany

**Keywords:** Elderly, Medical care, Claims data, Nursing homes, Nursing home residents

## Abstract

**Background:**

Elderly in need of long-term care tend to have worse health and have higher need of medical care than elderly without need for long-term care. Yet, characteristics associated with long-term care need can impede health care access: Higher levels of long-term care need come with physical and cognitive decline such as frailty and memory loss. Yet, it has not been investigated whether level of long-term care need is related to medical care utilization.

**Methods:**

We investigated the association between the level of long-term care and medical specialist utilization among nursing home residents and home care recipients. We applied zero-inflated Poisson regression with robust standard errors based on a sample of statutory health insurance members. The sample consisted of 100.000 elderly over age 60. We controlled for age, gender, morbidity and mortality, residential density, and general practitioner utilization.

**Results:**

We found a strong gradient effect of the level of long-term care for 9 out of 12 medical specialties: A higher level of long-term care need was associated with a lower probability of having a medical specialist visit. Yet, we did not find clear effects of the level of long-term care need on the intensity of medical specialist care. These findings were similar for both the nursing home and home care setting.

**Conclusion:**

The findings indicate that inequalities in medical specialist utilization exist between elderly with differing levels of long-term care need because differences in morbidity were controlled for. Elderly with higher need of long-term care might face more access barriers to specialist medical care.

## Background

Higher age is associated with increasing morbidity and higher health care utilization [1]. In industrialized countries, the majority of the elderly aged 60 years and over suffer from at least one chronic disease [2–4]. Consequently, elderly people show higher health care need and higher utilization of medical care than the overall population [5, 6].

However, previous studies have shown that those elderly who have need for long-term care tend to have unmet health care needs. For instance, elderly who live in nursing homes have less visits to medical specialists such as dentists, orthopedists, ophthalmologists and otorhinolaryngologist [7–12]. Consequently, if this lower level of medical utilization represents unmet care needs nursing home residents may be at risk of adverse outcomes, i.e. inadequate medication and therapy, avoidable hospitalizations, or falls [13–18].

The reasons for lower health care utilization of elderly in need of long-term care may lie in their physical and mental limitations. Frailty is an increasing phenomenon in the elderly population [19], and elderly in need of long-term care tend to be more frail than those without need of long-term care [20]. As a consequence, elderly in need of long-term care need more support in activities of daily living [21]. Moreover, elderly people in need of long-term care often suffer from cognitive impairment such as dementia [22–24]. Both frailty and cognitive impairment can increase the risk of unmet health care needs and perceive barriers to health care because the elderly may have more difficulties to assess their health care need, and to organize health care [25–28].

In Germany, about 90% of the population (i.e. 73 million people) are members of the statutory health insurance and therefore automatically also members of the statutory long-term care insurance. Only 10% of the population uses private health insurance and private long-term care insurance. Insured persons with physical and mental limitations can obtain long-term care benefits either in cash (to organize informal care by informal caregivers at their home), or in kind (to organize formal care at their home or long-term care in nursing homes). People with higher levels of long-term care obtain higher benefits from the long-term care insurance than people with lower levels of long-term care need. Benefits are granted irregardless of personal or household income or assets. However, benefits are capped, and statutorily insured persons have to pay the remainder if the costs of long-term care services exceed the granted benefits. The goal of these benefits is to provide insured persons with physical and mental limitations with support in activities of daily living. Therefore, long-term care services are distinct from medical services which aim to cure or prevent diseases.

Depending on the degree of physical and mental limitations and the according need for assistance in matters of daily life, care-dependent people are assigned a level of long-term care need. In Germany, until 2016, the level of long-term care need was called *Pflegestufe*. The Pflegestufe differentiated between the levels 1, 2, 3, and hardship cases. People in level 1 need assistance with daily activities (i.e. personal hygiene, mobility or eating) once a day, whereas people in level 2 need assistance three times a day. People in level 3 need several hours of assistance over the whole day. Hardship cases need at least 7 h of assistance during the day and at least 2 h of assistance during the night. In 2017, the Pflegestufe was replaced by the *Pflegegrad* which now differentiates between five care grades.

As elderly with a higher level of long-term care need are more limited in matters of daily life including the organization of medical care, they may have a higher risk of inadequate health care. However, although health care utilization of older people has been investigated [5, 29, 30], existing research on health care utilization of older people in need of long-term care is limited [31].

Consequently, the aim of this study is to focus on this particular group and to investigate whether differences in the utilization of 12 medical specialties exist between elderly in need of long-term care and elderly who are not in need of long-term care with a special focus on both the level of long-term care and the care setting. Such differences could indicate perceived access barriers and inequalities in medical care utilization among care-dependent elderly. In the following sections we describe our data source and statistical analysis followed by a description of our descriptive and multivariate findings. The findings are then discussed in the discussion section. Finally a conclusion is drawn.

## Methods

### Data source

We used claims data from the German health and long-term care insurance AOK (Allgemeine Ortskrankenkasse). The AOK consists of eleven regional health insurance funds which, taken together, represent the largest statutory health insurance fund in Germany. More than a third of the population who has statutory health insurance is covered by the AOK.

We drew a sample of 100,000 insured persons aged 60 years and over from the total AOK population. About 15% of the insured persons in this sample were in need of long-term care. This way, the sample represents the percentage of older people in need of long-term care among the total AOK population aged 60 years and over in Germany. Of these older people in need of long-term care, 5100 were nursing home residents and 9700 were community-dwelling elderly who received formal or informal home care. The insurance data included medical care visits, inpatient and outpatient diagnoses based on the German Modification of the International Classification of Diseases, 10th Revision, (ICD-10-GM), and sociodemographic characteristics (i.e. age, gender, and type of residential location).

### Statistical analysis

We applied zero-inflated Poisson regression with robust standard errors to model the distribution of medical care utilization. Zero-inflated Poisson regression accounts for non-normal distributions where there are many zero values. Although older people show high mean levels of health care utilization, there is considerable variation between non-morbid and multimorbid older people [5]. For instance, healthy elderly are more likely to have no medical care visits, and such zero-inflation is more likely to occur in medical specialist utilization or hospital utilization than in general practitioner utilization [32]. The zero-inflated Poisson model contains two components; the first part of the model predicts non-occurrence of a behavior, in our case the probability of not having any medical specialist visit. The second part of the model estimates how frequently the behavior occurred, i.e. the intensity of medical specialist care [33]. Yet, for two models (internist and orthopedics utilization among elderly with diagnosed motor impairment) a logistic regression was calculated because it fit the distribution of the data better than a zero-inflated Poisson.

The dependent variable was the overall visits to medical specialists in 2015. However, health insurance claims data do not reflect the actual number of visits but only those visits that were charged by physicians and were remunerated by statutory health insurance funds. Consequently, claims data document repeated visits to the same physician only once per quarter. However, if different physicians were contacted per quarter each visit was captured. We investigated 12 medical specialties. For each specialty, we included only those in a disease category that was relevant for the respective medical specialty under study. Multiple models were calculated per specialty when more than one diagnosis was relevant. Table 2 shows the resulting 45 models.

The main independent variable was combined from the level of long-term care (*Pflegestufe*) and the long-term care setting (nursing home vs. home care). We grouped the Pflegestufe into 3 levels of long-term care need (low = Pflegestufe 1, medium = Pflegestufe 2, and high = Pflegestufe 3 and hardship cases) and generated dummy variables for each of the 3 levels of long-term care need. Both long-term care settings were also defined as dummy variables and combined with the long-term care need. This resulted in 6 dummy variables differentiating between nursing home residents with low/medium/high long-term care need, and home care recipients with low/medium/high long-term care need. The reference group was older people without need of long-term care. These 6 dummy variables were included in all models; however, although both settings were included in the models simultaneously, we present each setting in a separate results table to fit on one page. Additional file 2 includes both settings.

We defined “older people in need of long-term care” as all people who are in need of long-term care according to § 14, German Social Code XI and who were assessed according to § 18, German Social Code XI. This means that the need of long-term care was legally assessed by the Medical Advisory Service of the German statutory health insurance funds (“Medizinischer Dienst der Krankenversicherung”). The assessment is based on nationwide guidelines and is conducted by trained nurses or physicians who observe and interview the persons in need of long-term care and relatives.

Control variables were age in 2015 (categorized into 7 groups), gender, mortality in 2015, overall visits to general practitioners in 2015, type of residential location (i. e. city, urban, and rural), and morbidity. We defined morbidity of each elderly based on diagnoses from the years 2014 and 2015 and categorized into 31 disease categories based on ICD-10 GM.

We investigated the stability of the findings by excluding the long-term care setting from the models; these models include only the covariates for long-term care need and are shown in Additional file 3. The results did not differ considerably from those presented in the results section.

## Results

### Descriptive findings

Table 1 shows that the elderly visit medical specialists on average between 0.3 and 2.1 times per year depending on the disease group and the type of medical specialty. The standard deviation of mostly 1.5 to 2.0 indicates that most elderly have between 0 and 4 annual visits to medical specialists. However, utilization ranges from 0 up until 35 visits. Further descriptive statistics on the characteristics of the sample are shown in Additional file 1.

**Table 1:** Descriptive statistics on specialist utilization among the elderly given a respective disease

Medical specialty	Disease category	Annual medical specialist utilization				
		Sample size	Mean visits	Minimum	Maximum	Standard deviation
Internal medicine	Renal failure	12,340	1.65	0	35	2.57
	Respiratory disease	18,303	1.56	0	32	2.21
	Heart disease	40,632	1.34	0	35	2.06
	Mono- and polyneuropathy	13,426	1.34	0	31	2.16
	Nutrition-related disease	17,016	1.28	0	31	2.07
	Cerebrovascular disease	14,389	1.25	0	31	2.05
	Coronary disease	32,416	1.21	0	32	2.00
	Intestinal disease	32,557	1.21	0	35	1.99
	Metabolic disorders	48,913	1.11	0	35	1.91
	Diabetes mellitus	30,683	1.11	0	32	1.96
	Thyroid disorders	23,589	1.11	0	35	1.92
	Parkinson’s disease	4887	1.10	0	30	2.01
	Arthropathy	43,937	1.06	0	35	1.87
	Hypertension	69,439	1.01	0	35	1.83
	Motor impairment	2533	0.83	0	14	1.57
	Palsy/paresis	2734	0.73	0	28	1.68
Cardiology	Heart disease	40,632	0.50	0	12	0.97
	Coronary disease	32,416	0.35	0	12	0.85
	Hypertension	69,439	0.31	0	12	0.78
Ophthalmology	Diseases of the eye	33,333	2.06	0	12	1.57
Orthopedy	Osteopathy and chondropathy	14,807	1.05	0	10	1.52
	Arthropathy	43,937	0.89	0	12	1.39
	Injury	13,313	0.89	0	10	1.42
	Spinal disease	46,093	0.87	0	12	1.37
	Motor impairment	2533	0.52	0	7	1.13
Gynecology	Disorders of female genital tract	9041	2.08	0	15	1.83
	Urinary tract disease	19,362	0.52	0	13	1.22
Urology	Prostate disease	11,666	1.80	0	16	1.72
	Urinary tract disease	19,362	1.11	0	11	1.63
Surgery	Injury	13,313	0.36	0	10	0.86
	Skin disease	12,848	0.26	0	10	0.74
Dermatology	Skin disease	12,848	1.26	0	9	1.50
	Bedsore/decubitus	6618	0.99	0	9	1.42
Otolaryngology	Diseases of the ear	18,325	1.27	0	10	1.33
Nephrology	Renal failure	12,340	0.53	0	31	1.58
Pneumology	Respiratory disease	18,303	0.57	0	9	1.17
Psychiatry / Neurology	Parkinson’s disease	4887	1.67	0	11	1.87
	Delusional/personality disorders	2925	1.65	0	10	1.92
	Dementia-related disease	10,807	1.22	0	10	1.72
	Palsy/paresis	2734	1.21	0	9	1.75
	Depression	18,477	0.99	0	11	1.60
	Neurosis	13,426	0.77	0	9	1.39
	Mono- and polyneuropathy	13,426	0.77	0	9	1.39
	Cerebrovascular disease	14,389	0.75	0	9	1.42
	Disorders due to psychoactive substance use	7162	0.58	0	9	1.31

The bivariate results in Table 2 indicate that there are clear differences in medical specialist utilization between elderly with and without need of long-term care. For instance, we find that among the elderly with a renal failure diagnosis, 57% of those with a low level of long-term care need did not have a visit to a physician of internal medicine. Among the elderly with a medium or high level of long-term care need 67 and 79%, respectively, did not have such a visit. In contrast, among the elderly with a renal failure diagnosis but without need of long-term care, only 44% did not have a visit to a physician of internal medicine. These patterns persist for all medical specialties except for urology and psychiatry/neurology; utilization of urologists is similar among elderly of all levels of long-term care. The probability of visiting neurologists or psychiatrists given a respective disease is higher among elderly with higher levels of long-term care.

**Table 2:** Descriptive statistics: Share of elderly with no medical specialist visit in 2015 by level of long-term care

Medical specialty	Disease categories	Elderly in need of long-term care						Elderly not in need of long-term care	
		Low level of care		Medium level of care		High level of care			
		Share	n	Share	n	%	n	Share	n
Internal medicine	Renal failure	57%	2040	67%	1483	79%	518	44%	8299
	Respiratory disease	56%	1929	68%	1236	78%	401	46%	14,737
	Heart disease	62%	4941	72%	3222	83%	1270	46%	31,199
	Mono- and polyneuropathy	59%	1709	67%	1044	75%	315	52%	10,358
	Nutrition-related disease	59%	1596	67%	907	77%	274	54%	14,239
	Cerebrovascular disease	67%	2106	76%	1790	84%	822	47%	9671
	Coronary disease	63%	3632	72%	2244	81%	843	54%	25,697
	Intestinal disease	63%	3057	74%	2118	86%	882	52%	26,500
	Metabolic disorders	63%	4110	72%	2670	83%	1059	57%	41,074
	Diabetes mellitus	64%	3338	73%	2260	82%	871	57%	24,214
	Thyroid disorders	62%	1983	73%	1153	85%	444	57%	20,009
	Parkinson’s disease	65%	743	74%	674	86%	383	54%	3087
	Arthropathy	65%	4184	74%	2505	83%	876	58%	36,372
	Hypertension	67%	6363	75%	4054	85%	1646	60%	57,376
	Motor impairment	76%	505	80%	402	88%	153	58%	1473
	Palsy/paresis	72%	575	80%	777	89%	393	61%	989
Cardiology	Heart disease	81%	4941	88%	3222	95%	1270	68%	31,199
	Coronary disease	83%	3632	89%	2244	96%	843	78%	25,697
	Hypertension	85%	6363	90%	4054	96%	1646	81%	57,376
Ophthalmology	Diseases of the eye	2%	3100	43%	1685	60%	591	16%	27,957
Orthopedics	Osteopathy and chondropathy	66%	2034	80%	1194	86%	466	53%	11,113
	Arthropathy	71%	4184	82%	2505	87%	876	58%	36,372
	Injury	70%	2017	83%	1629	89%	653	54%	9014
	Spinal disease	68%	3792	80%	2058	86%	721	59%	39,522
	Motor impairment	81%	505	89%	402	92%	153	70%	1473
Gynecology	Disorders of female genital tract	22%	1014	28%	456	32%	158	13%	16,454
	Urinary tract disease	88%	2861	94%	2830	96%	1408	72%	12,263
Urology	Prostate disease	21%	1564	20%	1228	21%	420	17%	20,120
	Urinary tract disease	74%	2861	73%	2830	78%	1408	51%	12,263
Surgery	Injury	83%	2017	86%	1629	90%	653	77%	9014
	Skin disease	85%	1244	88%	898	88%	898	84%	10,330
Dermatology	Skin disease	54%	1244	58%	898	57%	376	46%	10,330
	Bedsore/decubitus	68%	805	75%	849	83%	555	49%	4409
Otolaryngology	Diseases of the ear	47%	1853	50%	1056	59%	446	38%	14,970
Nephrology	Renal failure	83%	2040	88%	1483	92%	518	81%	8299
Pneumology	Respiratory disease	81%	1929	88%	1236	93%	401	73%	14,737
Psychiatry/ Neurology	Parkinson’s disease	45%	743	43%	674	39%	383	50%	3087
	Delusional/personality disorders	48%	464	43%	363	38%	233	54%	1865
	Dementia-related disease	60%	2470	54%	2538	53%	1504	66%	4295
	Palsy/paresis	62%	575	57%	777	52%	393	66%	989
	Depression	62%	2236	55%	1543	51%	681	69%	14,017
	Neurosis	64%	1553	57%	886	52%	306	72%	14,167
	Mono- and polyneuropathy	67%	1709	63%	1044	57%	315	71%	10,358
	Cerebrovascular disease	69%	2106	61%	1790	58%	822	76%	9671
	Disorders due to psychoactive substance use	67%	685	64%	388	47%	135	82%	5954

*Notes:* Sample size *n* = 100,000 insured persons of the AOK health and long-term care insurance fund

Low level = i.e. German „Pflegestufe 1″, medium level = „Pflegestufe 2″, high level = „Pflegestufe 3 “and hardship cases, disease categories are related to ICD-10-GM

### Multivariate findings

First, we found significant strong gradient effects of the level of long-term care on the probability of having no specialist visit for 8 out of 12 medical specialties, i.e. internal medicine, cardiology, ophthalmology, orthopedics, gynecology, nephrology, pneumology, psychiatry/neurology (Table 3 and Additional file 2: Table S2). In most of these cases (except for psychiatry/neurology), this means that higher levels of long-term care need were associated with a lower probability of having a medical specialist visit compared to the reference group without need of long-term care. For urology, surgery, dermatology, and otolaryngology, we cannot confirm a gradient effect because the majority of the effects was not significant. The largest gradient effects were found for the specialties cardiology, ophthalmology, and internal medicine given the respective disease (Table 3 and Additional file 2: Table S2).

**Table 3:**
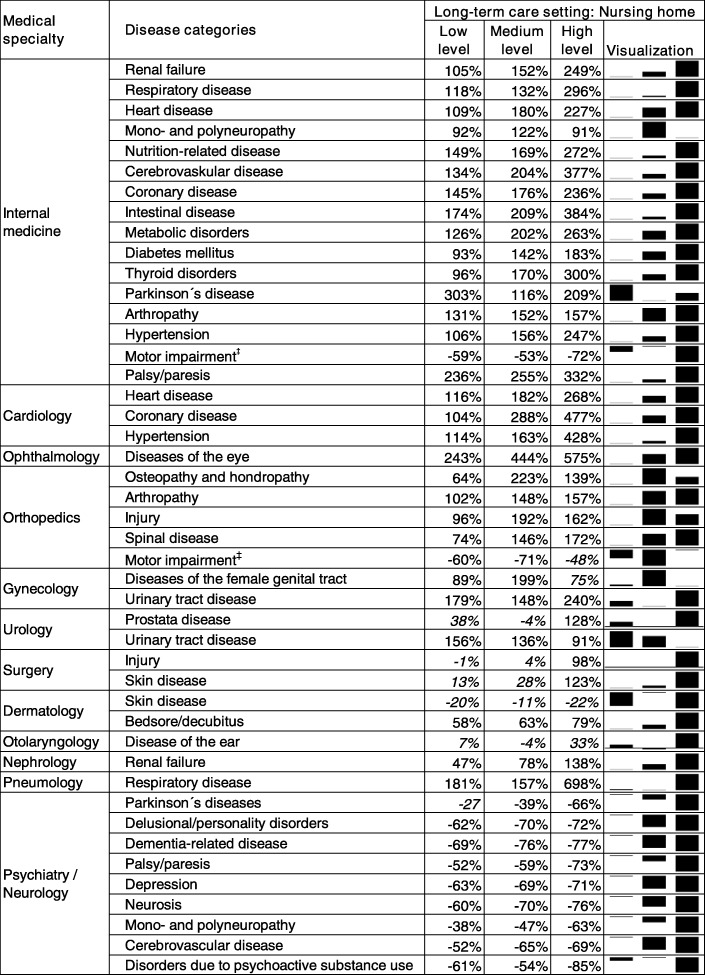
Nursing home residents: LTC need level and the risk of having no medical specialist visit

Notes: The table shows the percentage increase in risk of not having a specialist visit for nursing home residents (reference group: community-dwelling elderly without need of long-term care). Further covariates in the model: long-term care setting home care combined with the level of long term care need, gender, age, mortality, general practitioner visits, type of residential location and morbidity. Significant effects are printed bold, non-significant effects are printed italic

For instance, nursing home residents with a low long-term care level who have any of the investigated eye diseases have a 243% increase in risk of having no ophthalmology visit compared to the reference group, i.e. elderly with no need of long-term care. Nursing home residents with the highest long-term care level have a 575% increase in risk of seeing no ophthalmologist. The gradient effect is even larger in in the home care setting (Table 4): home care recipients with a low level of long-term care have a 136% increase in risk of having no ophthalmologist visit whereas those with the highest long-term care level have a 914% increase in risk compared to the reference group.

**Table 4:**
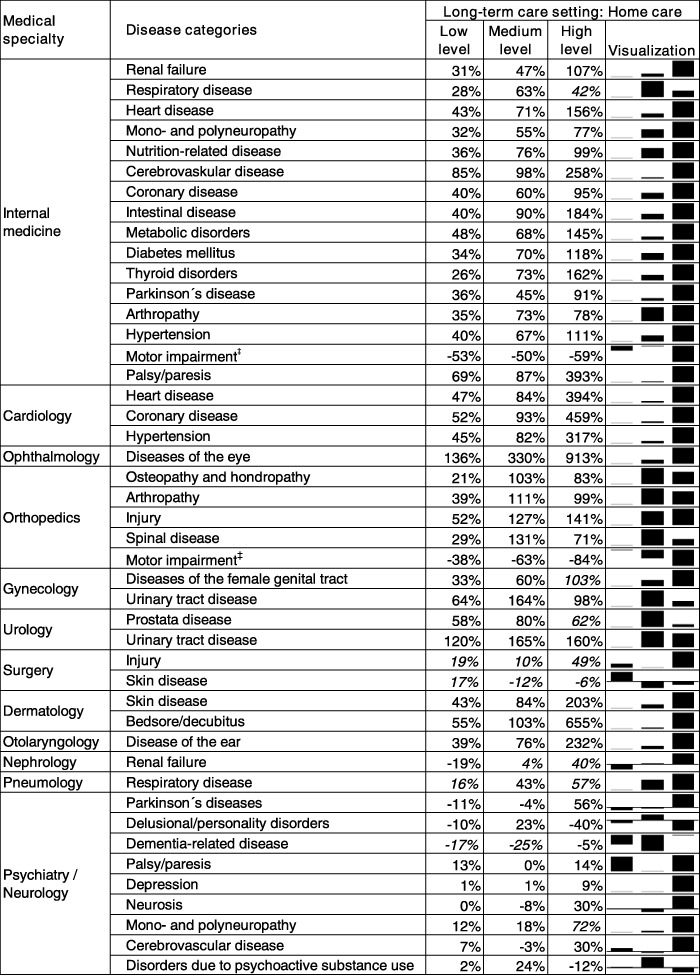
Home care recipients: LTC need level and the risk of having no medical specialist visit

Notes: The table shows the percentage increase in risk of not having a specialist visit for nursing home residents (reference group: community-dwelling elderly without need of long-term care). Further covariates in the model: long-term care setting home care combined with the level of long term care need, gender, age, mortality, general practitioner visits, type of residential location and morbidity. Significant effects are printed bold, non-significant effects are printed italic

For internal medicine, we found significant effects for all 16 investigated disease groups. However, for a few of these disease groups in certain settings, we could not confirm a consistent gradient effect with increasing levels of received long-term care (i. e. mono- and polyneuropathy, motor impairment and Parkinson’s disease among nursing home residents). For gynaecology, urology, and orthopedics we also found gradient effects but not for all of the investigated disease groups.

A reversed gradient effect was found for utilization of psychiatry/neurology: nursing home residents showed a lower probability of having no specialist visit than elderly without need of long-term care. There were hardly any effects of the level of long-term care on utilization of surgery: We found no significant effects among home care recipients, and no consistent effects among nursing home residents.

In all of these models, we controlled for General Practitioner (GP) utilization. There were however no or only small significant associations with medical specialist utilization. For some medical specialties, GP utilization was associated with a lower probability of having no specialist visit. This indicated no systematic substitution effects between GPs and medical specialists (see Additional file 4).

Second, for most medical specialties, we found no consistent gradient effect of the level of long-term care on the number of specialist visits (i.e. the intensity of specialist care) (Additional file 2: Table S2). Only in the case of neurologist/psychiatrist utilization, nursing home residents with higher levels of long-term care need tended to receive a higher intensity of medical care than those nursing home residents with lower levels of long-term care need.

Third, the effects of the level of long-term care were similar in both the home care and nursing home setting (Additional file 2: Table S2). We only found clear differences between the settings for the medical specialties dermatology, otolaryngology, and neurology/psychiatry. For dermatology, there were only small effects for nursing home residents but strong gradient effects for home care recipients. For otolaryngology, it was vice versa: we found no significant effects for home care recipients but strong gradient effects for nursing home residents. For neurology/psychiatry, nursing home residents showed a lower risk of having no specialist visit whereas the effects of the home care setting were rather inconsistent.

## Discussion

We gave an overview of medical specialist utilization of elderly people with differing levels of long-term care need. When controlling for differences in morbidity, we found that elderly with higher levels of long-term care were at a much higher risk of having no medical specialist visit than elderly who were not in need of long-term care. This finding applies to 8 out of 12 medical specialties, and the results were similar among the home care and the nursing home setting.

These findings support the importance of differentiating medical care utilization between elderly with and without need of long-term care. Although our descriptive findings showed that older people have on average at least one annual utilization to medical specialists, the picture is more nuanced. Our findings support previous studies that have already shown that elderly in need for long-term care have less visits to medical specialists, especially those living in nursing homes [9, 10]. Our results exceed previous findings showing that increasing levels of long-term care are associated with even lower specialist utilization. Although such kind of formal differentiation between different levels of long-term care need only exists in some countries so far [34–36], the implications of our findings may be cross-national: People who have limitations in the organization of daily activities may face barriers to medical care. The larger the limitations, the higher the risk of access barriers. One could argue that GPs might substitute the role of medical specialists among elderly in need of long-term care given the relatively high GP utilization of this group [9]. Yet, we controlled for GP utilization but did not find consistent or strong evidence for a relationship between GP visits and specialist visits (see Additional file 4): Having a GP visit either comes with a small decreased probability of not having a specialist visit or is unrelated to medical specialist utilization.

The gradient effects of the level of long-term care may be explained by objective or subjective access barriers. Especially elderly wheelchair users or homebound elderly face objective access barriers because in Germany, it is mostly GPs but not medical specialists who make (nursing) home visits. Subjective barriers to medical care also exist among elderly for instance feelings of shame in case of urinary incontinence [37]. Again other elderly perceive less need because of reduced health expectations. Contrary to younger people, older people perceive ageing as a natural, degenerative process not necessarily requiring a health care visit [38]. Also, they weigh the perceived value of medical care against the physical/mental burden of seeking care [25]. Yet, there is little evidence on the health care seeking process of elderly people in need of long-term care, especially of those living in nursing homes.

Although we found clear gradient effects on the risk of having no medical specialist visit, we did not find suchlike effects on the intensity of received specialist care. On the one hand, the intensity of received specialist care may mostly depend on the severity of disease which is not reflected in our disease categories. On the other hand, claims data do not capture the actual number of visits per person. Multiple visits to the same medical specialist per quarter are documented only as one visit. Only if a patient visits different medical specialists per quarter then each visit is documented. Therefore, the results on the intensity of specialist care have to be interpreted with caution.

Further limitations of our data concern the generalizability and the explanatory power of the models. Despite the fact that the AOK health and long-term care insurance fund covers large parts of the population in Germany, samples from statutory health insurance funds are selective and may not be representative of the overall population [39]. Also, the analyses were based on elderly with a diagnosis in the investigated disease category. Such a diagnosis requires a previous practitioner visit. Consequently, elderly without a practitioner visit, despite a respective disease - were not included in the analyses.

Moreover, the explanatory power of the analyzed models was relatively low ranging from 0.023 to 0.211 (see Additional file 4). Although the McFadden pseudo R^2^ measure generally produces lower coefficients than the R^2^ measure in linear regression analysis [40] our models do not include many of the factors that have been shown to explain health care utilization behavior [41–44]. For instance, health insurance claims data do not provide socioeconomic or demographic information which might have better explained differences in medical care utilization through differing health beliefs. Given the small range of covariates we did no systematic robustness test of our model.

## Conclusion

Our results indicate lower medical care utilization among elderly with higher levels of long-term care. However, although the underlying mechanisms of the findings have not been investigated so far, the findings imply that nursing care personnel and family relatives may not always be able to recognize the need for medical care among older care-dependent people. Furthermore, the findings indicate, that some elderly in need for long-term care may perceive physical or emotional barriers to medical specialists. Depending on the underlying mechanism, these findings may imply that long-term care provider and family relatives should be better trained in the assessment of medical care needs. Furthermore, the organization of and access to medical care should be improved, e.g. by organizing more home visits by medical specialists, and by better cooperation between GPs and medical specialists.

## Supplementary information

**Additional file 1.** Descriptive statistics of covariates.

**Additional file 2.** Zero-inflated Poisson Regression analysis: Associations between LTC setting, level of LTC need, and medical specialist utilization.

**Additional file 3.** Zero-inflated Poisson Regression analysis – stability analyses: Associations between level of LTC need and medical specialist utilization, without long-term care setting.

**Additional file 4.** Association between GP visits and medical specialist visits.

## Data Availability

The study is based on claims data that are located at the AOK Research Institute. These data are only available based upon a reasonable request and with permission of the AOK Research Institute.

## Abbreviations

AOK: AOK health and long-term care insurance (*Allgemeine Ortskrankenkasse)*
GP: General practitioner
ICD-10-GM: International Classification of Diseases, 10th Revision, German modification

## References

[1] Marengoni A, Angleman S, Melis R, Mangialasche F, Karp A, Garmen A (2011). Aging with multimorbidity: a systematic review of the literature. Ageing Res Rev.

[2] Collerton J, Jagger C, Yadegarfar ME, Davies K, Parker SG, Robinson L (2016). Deconstructing complex multimorbidity in the very old: findings from the Newcastle 85+ study. Biomed Res Int.

[3] Motel-Klingebiel A, Wurm S, Tesch-Römer C. Altern im Wandel: Befunde des Deutschen Alterssurveys (DEAS). Stuttgart: Kohlhammer Verlag; 2010.

[4] Kleina T, Horn A, Suhr R, Schaeffer D (2017). Zur Entwicklung der ärztlichen Versorgung in stationären Pflegeeinrichtungen–Ergebnisse einer empirischen Untersuchung. Das Gesundheitswesen.

[5] Bussche H, Schön G, Kolonko T, Hansen H, Wegscheider K, Glaeske G (2011). Patterns of ambulatory medical care utilization in elderly patients with special reference to chronic diseases and multimorbidity-results from a claims data based observational study in Germany. BMC Geriatr.

[6] Glynn LG, Valderas JM, Healy P, Burke E, Newell J, Gillespie P (2011). The prevalence of multimorbidity in primary care and its effect on health care utilization and cost. Fam Pract.

[7] Thibault L, Kergoat H (2016). Eye care services for older institutionalised individuals affected by cognitive and visual deficits: a systematic review. Ophthalmic Physiol Opt.

[8] Rothgang H, Müller R, Mundhenk R, Unger R (2014). BARMER GEK Pflegereport 2014. Schwerpunkt: Zahnärztliche Versorgung Pflegebedürftiger.

[9] Schmiemann Guido, Herget-Rosenthal Stefan, Hoffmann Falk (2015). Ärztliche Versorgung von Pflegeheimbewohnern. Zeitschrift für Gerontologie und Geriatrie.

[10] Balzer K, Butz S, Bentzel J, Boulkhemair D, Lühmann D (2013). Beschreibung und Bewertung der fachärztlichen Versorgung von Pflegeheimbewohnern in Deutschland. Schriftenreihe Health Technology Assessment Bd 125.

[11] Iliffe Steve, Davies Susan L., Gordon Adam L., Schneider Justine, Dening Tom, Bowman Clive, Gage Heather, Martin Finbarr C., Gladman John R.F., Victor Christina, Meyer Julienne, Goodman Claire (2015). Provision of NHS generalist and specialist services to care homes in England: review of surveys. Primary Health Care Research & Development.

[12] Rothgang H, Borchert L, Müller R, Unger R (2008). GEK Pflegereport 2008. Schwerpunktthema: Medizinische Versorgung in Pflegeheimen.

[13] Cox CA, van Jaarsveld HJ, Houterman S, van der Stegen JC, Wasylewicz AT, Grouls RJ (2016). Psychotropic drug prescription and the risk of falls in nursing home residents. J Am Med Dir Assoc.

[14] Ouslander JG, Lamb G, Perloe M, Givens JH, Kluge L, Rutland T (2010). Potentially avoidable hospitalizations of nursing home residents: frequency, causes, and costs. J Am Geriatr Soc.

[15] Lukas A, Mayer B, Onder G, Bernabei R, Denkinger MD (2015). Schmerztherapie in deutschen Pflegeeinrichtungen im europäischen Vergleich. Ergebnisse der SHELTER-Studie Schmerz.

[16] Johnell K (2015). Inappropriate drug use in people with cognitive impairment and dementia: a systematic review. Curr Clin Pharmacol.

[17] Morin L, Laroche M-L, Texier G, Johnell K (2016). Prevalence of potentially inappropriate medication use in older adults living in nursing homes: a systematic review. J Am Med Dir Assoc.

[18] Colloca G, Tosato M, Vetrano DL, Topinkova E, Fialova D, Gindin J (2012). Inappropriate drugs in elderly patients with severe cognitive impairment: results from the shelter study. PLoS One.

[19] Reeves D, Pye S, Ashcroft DM, Clegg A, Kontopantelis E, Blakeman T (2018). The challenge of ageing populations and patient frailty: can primary care adapt?. BMJ..

[20] Kojima G (2015). Prevalence of frailty in nursing homes: a systematic review and meta-analysis. J Am Med Dir Assoc.

[21] Björk S, Juthberg C, Lindkvist M, Wimo A, Sandman P-O, Winblad B (2016). Exploring the prevalence and variance of cognitive impairment, pain, neuropsychiatric symptoms and ADL dependency among persons living in nursing homes; a cross-sectional study. BMC Geriatr.

[22] Stock S, Ihle P, Simic D, Rupprecht C, Schubert I, Lappe V (2018). Prävalenz von Demenz bei Versicherten mit und ohne deutsche Staatsangehörigkeit. Bundesgesundheitsblatt-Gesundheitsforschung-Gesundheitsschutz..

[23] Prince M, Bryce R, Albanese E, Wimo A, Ribeiro W, Ferri CP (2013). The global prevalence of dementia: A systematic review and metaanalysis. Alzheimers Dement.

[24] Hoffmann F, Kaduszkiewicz H, Glaeske G, van den Bussche H, Koller D (2014). Prevalence of dementia in nursing home and community-dwelling older adults in Germany. Aging Clin Exp Res.

[25] Niesten D, van Mourik K, van der Sanden W (2013). The impact of frailty on oral care behavior of older people: a qualitative study. BMC Oral Health.

[26] Wu B, Plassman BL, Crout RJ, Liang J (2008). Cognitive function and oral health among community-dwelling older adults. J Gerontol Ser A Biol Med Sci.

[27] Cooper C, Lodwick R, Walters K, Raine R, Manthorpe J, Iliffe S (2017). Inequalities in receipt of mental and physical healthcare in people with dementia in the UK. Age Ageing.

[28] Burton LC, German PS, Gruber-Baldini AL, Hebel JR, Zimmerman S, Magaziner J (2001). Medical care for nursing home residents: differences by dementia status. Epidemiology of dementia in nursing homes research group. J Am Geriatr Soc.

[29] Bussche H, Kaduszkiewicz H, Schäfer I, Koller D, Hansen H, Scherer M (2016). Overutilization of ambulatory medical care in the elderly German population?–An empirical study based on national insurance claims data and a review of foreign studies. BMC Health Serv Res.

[30] Demographic change in the euro area: projections and consequences. European Central Bank Monthly Bulletin. 2006, 49–64.

[31] Balzer K, Butz S, Bentzel J, Boulkhemair D, Lühmann D (2013). Beschreibung und Bewertung der fachärztlichen Versorgung von Pflegeheimbewohnern in Deutschland. Schriftenreihe Health Technology Assessment Bd 125.

[32] Bähler C, Huber CA, Brüngger B, Reich O (2015). Multimorbidity, health care utilization and costs in an elderly community-dwelling population: a claims data based observational study. BMC Health Serv Res.

[33] Beaujean AA, Morgan GB (2016). Tutorial on using regression models with count outcomes using R. Pract Assess Res Eval.

[34] Tenand M, Bakx P, van Doorslaer E (2018). Long-term care use in the Netherlands: equal care for equal needs? An assessment using adminstrative data. mimeo.

[35] Iwagami M, Tamiya N (2019). The long-term care insurance system in Japan: past, present, and future. JMA J.

[36] Jeon B, Kwon S (2017). Health and long-term care systems for older people in the republic of Korea: policy challenges and lessons. Health Syst Reform.

[37] Vethanayagam N, Orrell A, Dahlberg L, McKee KJ, Orme S, Parker SG (2017). Understanding help-seeking in older people with urinary incontinence: an interview study. Health Soc Care Community.

[38] Horrocks S, Somerset M, Stoddart H, Peters TJ (2004). What prevents older people from seeking treatment for urinary incontinence? A qualitative exploration of barriers to the use of community continence services. Fam Pract.

[39] Hoffmann F, Icks A (2012). Structural differences between health insurance funds and their impact on health services research: results from the Bertelsmann health-care monitor. Gesundheitswesen..

[40] Scott LJ (1997). Regression models for categorical and limited dependent variables. Advanced quantitative techniques in the social sciences.

[41] Bremer D, Inhestern L, von dem Knesebeck O (2017). Social relationships and physician utilization among older adults—a systematic review. PLoS One.

[42] Hoebel J, Rattay P, Prütz F, Rommel A, Lampert T (2016). Socioeconomic status and use of outpatient medical care: the case of Germany. PLoS One.

[43] Hoebel J, Rommel A, Schröder LS, Fuchs J, Nowossadeck E, Lampert T (2017). Socioeconomic Inequalities in Health and Perceived Unmet Needs for Healthcare among the Elderly in Germany. Int J Environ Res Public Health.

[44] Terraneo M (2015). Inequities in health care utilization by people aged 50+: evidence from 12 European countries. Soc Sci Med.

