# Supplementary material for: Differences in medical specialist utilization among older people in need of long-term care – results from German health claims data
Source: Int J Equity Health. 2020 Feb 7;19:22. doi: 10.1186/s12939-020-1130-z (PMC7006141; doi:10.1186/s12939-020-1130-z)
Supplement: Supplementary file 1 — Additional file 1. Descriptive statistics of covariates. [file 12939_2020_1130_MOESM1_ESM.docx]

Additional file 1: Descriptive statistics of covariates

| **Variables** | | **N** | **Share** |
| --- | --- | --- | --- |
| Gender and age | Men, age 60-64 | 10,121 | 10.1 % |
|  | Men, age 65-69 | 8,196 | 8.2 % |
|  | Men, age 70-74 | 7,281 | 7.3 % |
|  | Men, age 75-79 | 8,523 | 8.5 % |
|  | Men, age 80-84 | 5,116 | 5.1 % |
|  | Men, age 85-89 | 2,503 | 2.5 % |
|  | Men, age 90+ | 8,60 | 0.9 % |
|  | Women, age 60-64 | 10,227 | 10.2 % |
|  | Women, age 65-69 | 8,841 | 8.8 % |
|  | Women, age 70-74 | 8,554 | 8.6 % |
|  | Women, age 75-79 | 11,384 | 11.4 % |
|  | Women, age 80-84 | 8,829 | 8.8 % |
|  | Women, age 85-89 | 5,982 | 6.0 % |
|  | Women, age 90+ | 3,583 | 3.6 % |
| Death | Person died during the year 2015 | 4,061 | 4.1 % |
| Type of residential location | Urban/city | 39,222 | 39.2 % |
|  | Town | 32,635 | 32.6 % |
|  | Rural | 28,143 | 28.1 % |
|  |  |  |  |
| Disease categories | Hypertension (I10-15) | 69,439 | 69.4 % |
|  | Metabolic disorders (E70-90) | 48,913 | 48.9 % |
|  | Spinal disease (M40-54) | 46,093 | 46.1 % |
|  | Arthropathy (M00-25) | 43,937 | 43.9 % |
|  | Heart disease (I20-52) | 40,632 | 40.6 % |
|  | Diseases of the eye (H00-59) | 33,333 | 33.3 % |
|  | Intestinal disease (K20-31; K40-46; K55-64) | 32,557 | 32.6 % |
|  | Coronary disease (I70-89) | 32,416 | 32.4 % |
|  | Diabetes mellitus (E10-14) | 30,683 | 30.7 % |
|  | Thyroid disorders (E00-07) | 23,589 | 23.6 % |
|  | Urinary Tract Disease (R30-39; N30-39) | 19,362 | 19.4 % |
|  | Depression (F30-39) | 18,477 | 18.5 % |
|  | Respiratory disease (J40-47) | 18,303 | 18.3 % |
|  | Diseases of the ear (H60-95) | 18,325 | 18.3 % |
|  | Nutrition-related disease (E40-46; E65-68) | 17,016 | 17.0 % |
|  | Neurosis (F40-48) | 16,912 | 16.9 % |
|  | Osteopathy and chondropathy (M80-94) | 14,807 | 14.8 % |
|  | Cerebrovascular diseases (I60-69) | 14,389 | 14.4 % |
|  | Mono- and polyneuropathy (G56-64) | 13,426 | 13.4 % |
|  | Injury (S00-99; T08-14) | 13,313 | 13.3 % |
|  | Skin disease (L20-30; C43-44) | 12,848 | 12.8 % |
|  | Renal failure (N17-19) | 12,340 | 12.3 % |
|  | Prostate disease (N40-51) | 11,666 | 11.7 % |
|  | Dementia-related disease (F00-09; G30-32) | 10,807 | 10.8 % |
|  | Disorders of female genital tract (N80-98) | 9,041 | 9.0 % |
|  | Disorders due to psychoactive substance use (F10-19) | 7,162 | 7.2 % |
|  | Bedsore/decubitus (L80-99) | 6,618 | 6.6 % |
|  | Parkinson’s disease (G20-26) | 4,887 | 4.9 % |
|  | Delusional/personality disorders (F20-29; 60-69) | 2,925 | 2.9 % |
|  | Palsy/paresis (G80-83) | 2,734 | 2.7 % |
|  | Motor impairment (U50-52) | 2,533 | 2.5 % |
|  | No disease diagnosed | 8,713 | 8.7 % |
|  |  |  |  |
| Level of long-term care need | Low level | 7,613 | 7.6 % |
|  | Medium level | 5,026 | 5.0 % |
|  | High level | 2,180 | 2.2 % |
| Long-term  care setting | Nursing home | 5,084 | 5.1 % |
|  | Home care | 9,735 | 9.7 % |

*Notes:* Sample size n=100,000 insured persons of the AOK health and long-term care insurance fund
Low level= i.e. German „Pflegestufe 1“, medium level =„Pflegestufe 2“, high level = „Pflegestufe 3“ and hardship cases, disease categories are related to ICD-10-GM (shown in parentheses)
