# Supplementary material for: Differences in medical specialist utilization among older people in need of long-term care – results from German health claims data
Source: Int J Equity Health. 2020 Feb 7;19:22. doi: 10.1186/s12939-020-1130-z (PMC7006141; doi:10.1186/s12939-020-1130-z)
Supplement: Supplementary file 2 — Additional file 2. Zero-inflated Poisson Regression analysis: Associations between LTC setting, level of LTC need, and medical specialist utilization. [file 12939_2020_1130_MOESM2_ESM.docx]

Additional File 2: Zero-inflated Poisson Regression analysis: Associations between LTC setting, level of LTC need, and medical specialist utilization

|  |  |  | **LTC setting: Nursing home** | | | | **LTC setting: Home care** | | | |
| --- | --- | --- | --- | --- | --- | --- | --- | --- | --- | --- |
| **Medical specialty** | **Disease category** | **LTC need level** | **% change in risk of having no visit** | **SE** | **% change in intensity of care** | **SE** | **% change in risk of having no visit** | **SE** | **% change in intensity of care** | **SE** |
| Internal medicine | Renal failure | Low level | 105.30*** | 0.14 | -8.09 | 0.11 | 31.50*** | 0.07 | 22.45*** | 0.04 |
|  |  | Medium level | 152.47*** | 0.14 | -4.78 | 0.14 | 46.78*** | 0.09 | 16.19*** | 0.06 |
|  |  | High level | 248.56*** | 0.21 | -8.24 | 0.16 | 106.64*** | 0.18 | 6.63 | 0.14 |
|  | Respiratory disease | Low level | 117.99*** | 0.15 | -19.82 | 0.12 | 27.70*** | 0.07 | 6.05 | 0.04 |
|  |  | Medium level | 132.23*** | 0.17 | -40.19*** | 0.13 | 62.66*** | 0.10 | 3.85 | 0.05 |
|  |  | High level | 295.62*** | 0.28 | -28.84 | 0.24 | 41.68 | 0.21 | -6.30 | 0.15 |
|  | Heart disease | Low level | 109.36*** | 0.10 | -15.44** | 0.08 | 42.86*** | 0.04 | 14.31*** | 0.03 |
|  |  | Medium level | 180.26*** | 0.10 | -11.37 | 0.11 | 71.39*** | 0.06 | 7.48 | 0.04 |
|  |  | High level | 226.64*** | 0.15 | -18.62 | 0.12 | 155.83*** | 0.13 | 8.18 | 0.11 |
|  | Mono- and polyneuropathy | Low level | 91.65*** | 0.19 | -23.02 | 0.15 | 31.56*** | 0.07 | 15.19*** | 0.04 |
|  |  | Medium level | 122.03*** | 0.20 | -36.20*** | 0.16 | 54.70*** | 0.10 | 11.88 | 0.06 |
|  |  | High level | 90.59** | 0.33 | -38.32** | 0.23 | 76.70*** | 0.21 | 31.34 | 0.14 |
|  | Nutrition-related disease | Low level | 148.83*** | 0.21 | -25.17** | 0.13 | 36.22*** | 0.07 | 16.04*** | 0.04 |
|  |  | Medium level | 168.84*** | 0.22 | -11.58 | 0.28 | 76.20*** | 0.12 | 8.47 | 0.07 |
|  |  | High level | 272.15*** | 0.30 | -6.73 | 0.20 | 99.26*** | 0.24 | 30.17 | 0.15 |
|  | Cerebrovascular disease | Low level | 133.97*** | 0.16 | -28.22*** | 0.11 | 85.23*** | 0.07 | 10.69** | 0.05 |
|  |  | Medium level | 204.45*** | 0.15 | -27.57** | 0.13 | 98.17*** | 0.10 | -9.03 | 0.06 |
|  |  | High level | 376.72*** | 0.19 | -11.43 | 0.15 | 258.10*** | 0.18 | -6.32 | 0.15 |
|  | Coronary disease | Low level | 144.59*** | 0.12 | -27.01** | 0.10 | 52.19*** | 0.05 | -7.79 | 0.03 |
|  |  | Medium level | 175.78*** | 0.15 | -26.51 | 0.17 | 92.83*** | 0.08 | -4.87 | 0.05 |
|  |  | High level | 235.63*** | 0.20 | -4.88 | 0.15 | 458.65*** | 0.15 | 29.89 | 0.11 |
|  | Intestinal disease | Low level | 173.89*** | 0.12 | -1.20 | 0.10 | 40.43*** | 0.06 | 9.99*** | 0.03 |
|  |  | Medium level | 209.08*** | 0.14 | -17.43 | 0.16 | 90.25*** | 0.08 | 10.03 | 0.05 |
|  |  | High level | 383.84*** | 0.18 | -2.86 | 0.13 | 183.94*** | 0.16 | 3.52 | 0.13 |
|  | Metabolic disorders | Low level | 125.59*** | 0.12 | -18.65** | 0.10 | 48.11*** | 0.05 | 14.75*** | 0.03 |
|  |  | Medium level | 201.54*** | 0.13 | -18.06 | 0.14 | 68.10*** | 0.07 | 7.88 | 0.04 |
|  |  | High level | 262.82*** | 0.17 | -17.63 | 0.14 | 145.22*** | 0.14 | 7.38 | 0.11 |
|  | Diabetes mellitus | Low level | 93.00*** | 0.13 | -16.10 | 0.11 | 33.57*** | 0.05 | 16.28*** | 0.30 |
|  |  | Medium level | 141.72*** | 0.14 | -15.58 | 0.16 | 69.93*** | 0.08 | 12.08** | 0.05 |
|  |  | High level | 183.30*** | 0.20 | -22.84 | 0.16 | 118.10*** | 0.15 | 18.52 | 0.11 |
|  | Thyroid disorders | Low level | 95.88*** | 0.16 | -2.27 | 0.13 | 26.41*** | 0.07 | 10.56** | 0.04 |
|  |  | Medium level | 169.97*** | 0.20 | -12.69 | 0.26 | 73.39*** | 0.11 | 14.63 | 0.08 |
|  |  | High level | 299.78*** | 0.28 | -18.04 | 0.19 | 162.29*** | 0.22 | 39.48 | 0.17 |
|  | Parkinson´s disease | Low level | 302.57*** | 0.28 | -5.70 | 0.20 | 35.54** | 0.12 | 14.56** | 0.07 |
|  |  | Medium level | 115.88* | 0.35 | -14.10 | 0.47 | 44.52** | 0.15 | 2.95 | 0.08 |
|  |  | High level | 208.96*** | 0.26 | 33.04 | 0.17 | 90.97** | 0.30 | -3.41 | 0.24 |
|  | Arthropathy | Low level | 130.82*** | 0.12 | -14.44 | 0.10 | 35.33*** | 0.05 | 10.43*** | 0.03 |
|  |  | Medium level | 152.23*** | 0.14 | -19.53 | 0.15 | 73.22*** | 0.07 | 18.93*** | 0.05 |
|  |  | High level | 157.28*** | 0.27 | -37.85 | 0.24 | 77.76*** | 0.16 | 3.18 | 0.13 |
|  | Hypertension | Low level | 106.09*** | 0.10 | -14.75** | 0.08 | 40.10*** | 0.04 | 10.69*** | 0.03 |
|  |  | Medium level | 156.02*** | 0.11 | -20.02** | 0.11 | 67.40*** | 0.06 | 5.86 | 0.04 |
|  |  | High level | 247.06*** | 0.15 | -20.97 | 0.12 | 110.57*** | 0.12 | 7.55 | 0.10 |
|  | Motor impairment^‡^ | Low level | -59.46*** | 0.26 | - | - | -52.81*** | 0.15 | - | - |
|  |  | Medium level | -53.07*** | 0.26 | - | - | -50.29*** | 0.21 | - | - |
|  |  | High level | -71.73*** | 0.38 | - | - | -58.72** | 0.43 | - | - |
|  | Palsy/paresis | Low level | 236.44*** | 0.35 | -15.36 | 0.25 | 68.88*** | 0.15 | 23.02 | 0.11 |
|  |  | Medium level | 254.99*** | 0.26 | -10.74 | 0.25 | 87.29*** | 0.17 | -15.12 | 0.12 |
|  |  | High level | 331.87*** | 0.35 | -24.45 | 0.33 | 392.80*** | 0.30 | 18.19 | 0.22 |
| Cardiology | Heart disease | Low level | 115.93*** | 0.15 | -22.86** | 0.11 | 47.18*** | 0.06 | -5.22 | 0.05 |
|  |  | Medium level | 181.62*** | 0.17 | -25.09** | 0.15 | 84.13*** | 0.10 | -9.26 | 0.07 |
|  |  | High level | 268.50*** | 0.38 | -36.88 | 0.39 | 393.96*** | 0.24 | 8.54 | 0.24 |
|  | Coronary disease | Low level | 104.47*** | 0.21 | -27.01** | 0.15 | 52.19*** | 0.08 | -7.79 | 0.06 |
|  |  | Medium level | 288.37*** | 0.23 | -26.51 | 0.20 | 92.83*** | 0.12 | -4.87 | 0.09 |
|  |  | High level | 476.60*** | 0.41 | -12.57 | 0.46 | 317.11*** | 0.27 | 12.36 | 0.21 |
|  | Hypertension | Low level | 113.75*** | 0.15 | -27.01** | 0.12 | 52.19*** | 0.07 | -7.79 | 0.05 |
|  |  | Medium level | 163.18*** | 0.18 | -26.51 | 0.15 | 92.83*** | 0.10 | -4.87 | 0.07 |
|  |  | High level | 427.91*** | 0.31 | -4.88 | 0.33 | 458.65*** | 0.24 | 29.89 | 0.24 |
| Ophthalmology | Diseases of the eye | Low level | 243.41*** | 0.17 | -5.87 | 0.04 | 135.69*** | 0.10 | -2.54 | 0.02 |
|  |  | Medium level | 444.17*** | 0.16 | -0.36 | 0.05 | 330.33*** | 0.13 | -4.07 | 0.03 |
|  |  | High level | 575.15*** | 0.21 | -6.95 | 0.08 | 913.47*** | 0.20 | -19.06** | 0.09 |

| **continued** | | | | | | | | | | |
| --- | --- | --- | --- | --- | --- | --- | --- | --- | --- | --- |
|  |  |  | **LTC setting: Nursing home** | | | | **LTC setting: Home care** | | | |
| **Medical specialty** | **Disease category** | **LTC nedd level** | **% change in risk of having no visit** | **SE** | **% change in intensity of care** | **SE** | **% change in risk of having no visit** | **SE** | **% change in intensity of care** | **SE** |
| Orthopedics | Osteopathy and chondropathy | Low level | 64.45*** | 0.18 | -30.86*** | 0.11 | 21.45** | 0.08 | -9.41*** | 0.03 |
|  |  | Medium level | 222.59*** | 0.16 | -4.45 | 0.11 | 103.22*** | 0.12 | -7.44 | 0.07 |
|  |  | High level | 138.68*** | 0.26 | -29.32 | 0.19 | 82.69** | 0.26 | -39.42*** | 0.19 |
|  | Arthropathy | Low level | 101.67*** | 0.12 | -20.58*** | 0.09 | 39.30*** | 0.05 | -4.86 | 0.03 |
|  |  | Medium level | 147.94*** | 0.12 | -7.79 | 0.10 | 111.28*** | 0.08 | -8.12 | 0.05 |
|  |  | High level | 156.68*** | 0.18 | -2.39 | 0.13 | 99.27*** | 0.22 | -33.37** | 0.17 |
|  | Injury | Low level | 95.57*** | 0.17 | -21.99** | 0.12 | 52.42*** | 0.08 | 1.36 | 0.04 |
|  |  | Medium level | 192.01*** | 0.15 | -5.17 | 0.11 | 127.16*** | 0.13 | -12.17 | 0.08 |
|  |  | High level | 161.51*** | 0.23 | -8.16 | 0.18 | 141.12*** | 0.31 | -37.13 | 0.24 |
|  | Spinal disease | Low level | 73.90*** | 0.13 | -24.66*** | 0.09 | 29.31*** | 0.05 | -6.81*** | 0.03 |
|  |  | Medium level | 145.77*** | 0.13 | -9.65 | 0.10 | 131.21*** | 0.09 | -8.39 | 0.05 |
|  |  | High level | 172.19*** | 0.21 | -19.13 | 0.15 | 70.81** | 0.24 | -43.50*** | 0.17 |
|  | Motor impairment^‡^ | Low level | -60.25*** | 0.31 | - | - | -38.10*** | 0.17 | - | - |
|  |  | Medium level | -71.21*** | 0.30 | - | - | -62.61*** | 0.26 | - | - |
|  |  | High level | -48.28 | 0.41 | - | - | -83.63 | 0.67 | - | - |
| Gynecology | Diseases of the female genital tract | Low level | 88.69** | 0.31 | -11.39 | 0.15 | 33.32** | 0.13 | -0.66 | 0.04 |
|  |  | Medium level | 198.62*** | 0.30 | 18.31 | 0.10 | 59.98** | 0.22 | 2.92 | 0.07 |
|  |  | High level | 75.38 | 0.54 | -26.94 | 0.25 | 103.19 | 0.41 | -8.09 | 0.18 |
|  | Urinary tract disease | Low level | 178.67*** | 0.19 | -5.91 | 0.12 | 63.63*** | 0.10 | -3.01 | 0.05 |
|  |  | Medium level | 147.94*** | 0.20 | -17.72 | 0.16 | 164.36*** | 0.13 | 2.61 | 0.08 |
|  |  | High level | 240.38*** | 0.29 | -33.32 | 0.24 | 98.12** | 0.28 | -30.06 | 0.20 |
| Urology | Prostata disease | Low level | 37.76 | 0.25 | 9.68 | 0.06 | 58.23*** | 0.12 | -0.78 | 0.03 |
|  |  | Medium level | -3.60 | 0.29 | -0.43 | 0.06 | 80.27*** | 0.15 | 0.74 | 0.04 |
|  |  | High level | 128.31*** | 0.27 | -5.94 | 0.07 | 62.02 | 0.27 | 19.14*** | 0.06 |
|  | Urinary tract disease | Low level | 155.83*** | 0.21 | 5.19 | 0.07 | 120.32*** | 0.13 | 0.10 | 0.04 |
|  |  | Medium level | 135.97*** | 0.17 | 6.57 | 0.05 | 164.96*** | 0.14 | 5.62 | 0.04 |
|  |  | High level | 90.98*** | 0.23 | -7.54 | 0.07 | 159.63*** | 0.19 | 22.82*** | 0.06 |
| Surgery | Injury | Low level | -1.20 | 0.22 | -22.40 | 0.17 | 19.49 | 0.11 | 8.45 | 0.09 |
|  |  | Medium level | 4.19 | 0.23 | -25.77 | 0.18 | 9.90 | 0.17 | -20.84 | 0.13 |
|  |  | High level | 97.74*** | 0.24 | 17.86 | 0.18 | 49.42 | 0.36 | -20.34 | 0.29 |
|  | Skin disease | Low level | 13.31 | 0.29 | 2.01 | 0.26 | 16.84 | 0.14 | 18.08 | 0.12 |
|  |  | Medium level | 27.63 | 0.28 | -10.50 | 0.24 | -11.89 | 0.38 | -31.27 | 0.31 |
|  |  | High level | 123.19*** | 0.29 | 115.17*** | 0.24 | -5.95 | 0.54 | -42.71 | 0.40 |
| Dermatology | Skin disease | Low level | -19.96 | 0.17 | 0.31 | 0.06 | 43.32*** | 0.09 | -0.53 | 0.04 |
|  |  | Medium level | -11.05 | 0.16 | 9.97 | 0.06 | 84.48*** | 0.14 | -16.72** | 0.07 |
|  |  | High level | -21.60 | 0.21 | 12.20 | 0.07 | 202.91*** | 0.31 | -28.10** | 0.16 |
|  | Bedsore/decubitus | Low level | 57.73** | 0.21 | 2.56 | 0.10 | 54.54*** | 0.15 | -7.38 | 0.07 |
|  |  | Medium level | 63.39*** | 0.19 | 3.73 | 0.09 | 103.34*** | 0.19 | -17.83 | 0.12 |
|  |  | High level | 79.34*** | 0.23 | 6.23 | 0.11 | 655.21*** | 0.31 | -12.61 | 0.18 |
| Otolaryngology | Disease of the ear | Low level | 7.31 | 0.18 | 2.47 | 0.06 | 39.09*** | 0.09 | -4.57 | 0.03 |
|  |  | Medium level | -3.57 | 0.17 | 12.67** | 0.05 | 76.22*** | 0.15 | -10.23 | 0.06 |
|  |  | High level | 32.57 | 0.19 | 15.00** | 0.07 | 232.36*** | 0.27 | -20.80 | 0.13 |
| Nephrology | Renal failure | Low level | 47.42** | 0.19 | 30.33 | 0.19 | -18.59** | 0.08 | 29.64*** | 0.06 |
|  |  | Medium level | 77.75*** | 0.18 | 22.10 | 0.25 | 3.78 | 0.11 | 42.48*** | 0.09 |
|  |  | High level | 138.18*** | 0.30 | 38.15 | 0.21 | 40.21 | 0.23 | 55.10** | 0.20 |
| Pneumology | Respiratory disease | Low level | 181.45*** | 0.23 | -16.72 | 0.20 | 16.40 | 0.08 | 9.34 | 0.05 |
|  |  | Medium level | 156.90*** | 0.25 | -32.20** | 0.19 | 43.11*** | 0.12 | 4.71 | 0.09 |
|  |  | High level | 697.76*** | 0.72 | -27.50 | 0.90 | 57.20 | 0.26 | 15.67 | 0.23 |
| Psychiatry / Neurology | Parkinson´s diseases | Low level | -27.23 | 0.21 | 7.71 | 0.05 | -10.86 | 0.11 | 5.48 | 0.03 |
|  |  | Medium level | -39.05** | 0.19 | 15.13*** | 0.04 | -3.84 | 0.14 | 11.70*** | 0.03 |
|  |  | High level | -66.38*** | 0.24 | 23.55*** | 0.05 | 55.76 | 0.23 | 7.44 | 0.08 |
|  | Delusional/ personality disorders | Low level | -61.90*** | 0.22 | 8.11 | 0.05 | -9.99 | 0.16 | -5.03 | 0.05 |
|  |  | Medium level | -69.64*** | 0.23 | 14.85*** | 0.05 | 22.70 | 0.23 | 3.08 | 0.07 |
|  |  | High level | -72.17*** | 0.27 | 18.08*** | 0.06 | -39.59 | 0.35 | 5.29 | 0.08 |
|  | Dementia-related disease | Low level | -69.10*** | 0.09 | 15.52*** | 0.03 | -16.72** | 0.07 | 6.95** | 0.03 |
|  |  | Medium level | -75.67*** | 0.09 | 20.16*** | 0.03 | -24.68*** | 0.09 | 7.12** | 0.03 |
|  |  | High level | -77.44*** | 0.10 | 27.72*** | 0.03 | -5.06 | 0.14 | 8.84 | 0.05 |
|  | Palsy/paresis | Low level | -52.14*** | 0.26 | 19.87*** | 0.09 | 13.21 | 0.15 | 25.78*** | 0.06 |
|  |  | Medium level | -59.16*** | 0.19 | 36.51*** | 0.06 | 0.37 | 0.16 | 23.76*** | 0.06 |
|  |  | High level | -72.74*** | 0.23 | 34.77*** | 0.07 | 14.49 | 0.25 | 18.09 | 0.09 |
|  | Depression | Low level | -63.29*** | 0.12 | 14.95*** | 0.04 | 0.83 | 0.07 | 4.09 | 0.03 |
|  |  | Medium level | -69.29*** | 0.12 | 20.08*** | 0.03 | 0.59 | 0.10 | 7.92** | 0.03 |
|  |  | High level | -70.95*** | 0.15 | 23.00*** | 0.04 | 9.10 | 0.19 | 0.48 | 0.07 |
|  | Neurosis | Low level | -60.32*** | 0.16 | 7.02 | 0.05 | 0.30 | 0.09 | 2.67 | 0.03 |
|  |  | Medium level | -70.29*** | 0.17 | 15.10*** | 0.04 | -8.30 | 0.13 | 3.13 | 0.05 |
|  |  | High level | -75.72*** | 0.23 | 23.66*** | 0.05 | 29.85 | 0.29 | -4.62 | 0.11 |
|  | Mono- and polyneuropathy | Low level | -37.77*** | 0.16 | 22.17*** | 0.06 | 12.04 | 0.08 | 6.37 | 0.03 |
|  |  | Medium level | -46.55*** | 0.15 | 20.12*** | 0.05 | 18.31 | 0.12 | 8.86 | 0.05 |
|  |  | High level | -63.37*** | 0.25 | 24.64*** | 0.07 | 72.47** | 0.25 | 11.13 | 0.10 |
|  | Cerebrovascular disease | Low level | -51.88*** | 0.13 | 21.78*** | 0.04 | 6.77 | 0.08 | 10.44*** | 0.03 |
|  |  | Medium level | -65.37*** | 0.12 | 19.70*** | 0.04 | -3.00 | 0.10 | 9.34** | 0.04 |
|  |  | High level | -68.70*** | 0.14 | 31.09*** | 0.04 | 30.07 | 0.18 | 12.83 | 0.06 |
|  | Disorders due to psychoactive substance use | Low level | -60.66*** | 0.20 | 38.12*** | 0.07 | 1.74 | 0.16 | -3.48 | 0.06 |
|  |  | Medium level | -54.04*** | 0.25 | 16.61** | 0.08 | 24.48 | 0.25 | 15.67 | 0.09 |
|  |  | High level | -85.08*** | 0.40 | 12.29 | 0.08 | -11.82 | 0.49 | 49.00*** | 0.15 |

*Notes:* Alpha level: *** = 0.01. ** = 0.05. * = 0.1; shown are percentage change in risk/intensity of care, SE = standard error
Low level= i.e. German „Pflegestufe 1“, medium level =„Pflegestufe 2“, high level = „Pflegestufe 3“ and hardship cases; LTC level = long-term care level; reference group = elderly not in need of long-term care

Further covariates in the model: gender, age, mortality, general practitioner visits, type of residential location and morbidity

^‡^based on logistic regression analysis
