# Supplementary material for: Differences in medical specialist utilization among older people in need of long-term care – results from German health claims data
Source: Int J Equity Health. 2020 Feb 7;19:22. doi: 10.1186/s12939-020-1130-z (PMC7006141; doi:10.1186/s12939-020-1130-z)
Supplement: Supplementary file 3 — Additional file 3. Zero-inflated Poisson Regression analysis – stability analyses: Associations between level of LTC need and medical specialist utilization, without long-term care setting. [file 12939_2020_1130_MOESM3_ESM.docx]

Additional File 3: Stability analyses of the effect of the level of long-term care need (without long-term care setting)

| **Medical specialty** | **Disease category** | **LTC need level** | **Having no visit** | | **Intensity of care** | |
| --- | --- | --- | --- | --- | --- | --- |
|  |  |  | **% change** | **SE** | **% change** | **SE** |
| Internal medicine | Renal failure | Low level | 41.42*** | 0.06 | 19.58*** | 0.04 |
|  |  | Medium level | 72.60*** | 0.08 | 12.54** | 0.06 |
|  |  | High level | 144.78*** | 0.14 | 4.34 | 0.11 |
|  | Respiratory disease | Low level | 39.24*** | 0.06 | 3.97 | 0.03 |
|  |  | Medium level | 83.93*** | 0.09 | -3.64 | 0.05 |
|  |  | High level | 109.51*** | 0.16 | -9.37 | 0.13 |
|  | Heart disease | Low level | 52.29*** | 0.04 | 11.47*** | 0.03 |
|  |  | Medium level | 99.24*** | 0.06 | 4.28 | 0.04 |
|  |  | High level | 178.38*** | 0.10 | 0.43 | 0.09 |
|  | Mono- and polyneuropathy | Low level | 39.97*** | 0.07 | 12.28*** | 0.04 |
|  |  | Medium level | 75.54*** | 0.09 | 4.56 | 0.06 |
|  |  | High level | 92.69*** | 0.17 | 13.08 | 0.13 |
|  | Nutrition-related disease | Low level | 47.12*** | 0.07 | 13.18*** | 0.04 |
|  |  | Medium level | 95.13*** | 0.10 | 5.42 | 0.07 |
|  |  | High level | 149.62*** | 0.19 | 23.20 | 0.13 |
|  | Cerebrovascular disease | Low level | 94.94*** | 0.07 | 5.97 | 0.05 |
|  |  | Medium level | 130.25*** | 0.09 | -12.86** | 0.06 |
|  |  | High level | 294.31*** | 0.14 | -6.22 | 0.11 |
|  | Coronary disease | Low level | 58.92*** | 0.08 | -9.62 | 0.06 |
|  |  | Medium level | 136.42*** | 0.11 | -8.31 | 0.08 |
|  |  | High level | 451.97*** | 0.23 | 16.49 | 0.23 |
|  | Intestinal disease | Low level | 55.81*** | 0.05 | 9.10*** | 0.03 |
|  |  | Medium level | 122.02*** | 0.07 | 4.33 | 0.05 |
|  |  | High level | 243.59*** | 0.13 | 2.60 | 0.10 |
|  | Metabolic disorders | Low level | 58.05*** | 0.05 | 11.80*** | 0.03 |
|  |  | Medium level | 101.94*** | 0.06 | 3.59 | 0.04 |
|  |  | High level | 182.86*** | 0.11 | 1.52 | 0.09 |
|  | Diabetes mellitus | Low level | 41.64*** | 0.05 | 13.51*** | 0.03 |
|  |  | Medium level | 89.21*** | 0.07 | 7.43 | 0.05 |
|  |  | High level | 142.84*** | 0.12 | 7.48 | 0.10 |
|  | Thyroid disorders | Low level | 34.61*** | 0.07 | 9.63** | 0.04 |
|  |  | Medium level | 98.19*** | 0.10 | 9.12 | 0.08 |
|  |  | High level | 221.29*** | 0.17 | 22.76 | 0.15 |
|  | Parkinson´s disease | Low level | 58.11*** | 0.12 | 13.42 | 0.07 |
|  |  | Medium level | 56.32*** | 0.14 | -0.09 | 0.11 |
|  |  | High level | 122.84*** | 0.21 | 13.08 | 0.15 |
|  | Arthropathy | Low level | 45.67*** | 0.05 | 8.72*** | 0.03 |
|  |  | Medium level | 95.45*** | 0.06 | 12.10** | 0.05 |
|  |  | High level | 113.01*** | 0.13 | -5.50 | 0.11 |
|  | Hypertension | Low level | 53.36*** | 0.06 | 8.35*** | 0.03 |
|  |  | Medium level | 105.13*** | 0.09 | 0.88 | 0.04 |
|  |  | High level | 352.87*** | 0.19 | 0.43 | 0.08 |
|  | Motor impairment‡ | Low level | -54.22*** | 0.14 | - | - |
|  |  | Medium level | -50.74*** | 0.18 | - | - |
|  |  | High level | -66.56*** | 0.30 | - | - |
|  | Palsy/paresis | Low level | 87.35*** | 0.14 | 20.07 | 0.10 |
|  |  | Medium level | 119.80*** | 0.16 | -12.91 | 0.12 |
|  |  | High level | 334.55*** | 0.23 | 3.84 | 0.20 |
| Cardiology | Heart disease | Low level | 56.26*** | 0.06 | -6.81 | 0.04 |
|  |  | Medium level | 109.69*** | 0.09 | -12.26** | 0.07 |
|  |  | High level | 332.22*** | 0.20 | -11.00 | 0.20 |
|  | Coronary disease | Low level | 58.92*** | 0.08 | -9.62 | 0.06 |
|  |  | Medium level | 136.42*** | 0.11 | -8.31 | 0.08 |
|  |  | High level | 352.87*** | 0.19 | 2.75 | 0.20 |
|  | Hypertension | Low level | 58.92*** | 0.08 | -8.24 | 0.05 |
|  |  | Medium level | 136.42*** | 0.11 | -14.89** | 0.07 |
|  |  | High level | 451.97*** | 0.23 | 2.75 | 0.20 |
| Ophthalmology | Diseases of the eye | Low level | 151.80*** | 0.10 | -3.09 | 0.02 |
|  |  | Medium level | 357.72*** | 0.12 | -2.96 | 0.03 |
|  |  | High level | 666.20*** | 0.16 | -13.11** | 0.06 |

| **continued** | | | | | | |
| --- | --- | --- | --- | --- | --- | --- |
| **Medical specialty** | **Disease category** | **LTC need level** | **Having no visit** | | **Intensity of care** | |
|  |  |  | **% change** | **SE** | **% change** | **SE** |
| Orthopedics | Osteopathy and chondropathy | Low level | 27.59*** | 0.07 | -11.74** | 0.03 |
|  |  | Medium level | 134.64*** | 0.10 | -5.88 | 0.06 |
|  |  | High level | 99.17*** | 0.19 | -33.88*** | 0.14 |
|  | Arthropathy | Low level | 46.2*** | 0.05 | -6.47** | 0.03 |
|  |  | Medium level | 130.58*** | 0.07 | -7.78* | 0.05 |
|  |  | High level | 127.46*** | 0.14 | -16.36* | 0.11 |
|  | Injury | Low level | 59.31*** | 0.08 | -1.77 | 0.04 |
|  |  | Medium level | 131.36*** | 0.11 | -8.99 | 0.06 |
|  |  | High level | 155.75*** | 0.19 | -19.76 | 0.15 |
|  | Spinal disease | Low level | 38.14*** | 0.05 | -8.47*** | 0.03 |
|  |  | Medium level | 132.22*** | 0.08 | -8.36 | 0.05 |
|  |  | High level | 107.85*** | 0.16 | -31.09*** | 0.12 |
|  | Motor impairment^‡^ | Low level | -42.79*** | 0.16 | - | - |
|  |  | Medium level | -65.81*** | 0.22 | - | - |
|  |  | High level | -58.37*** | 0.36 | - | - |
| Gynecology | Diseases of the female genital tract | Low level | 38.81*** | 0.12 | -1.80 | 0.04 |
|  |  | Medium level | 91.21*** | 0.18 | 7.00 | 0.06 |
|  |  | High level | 83.29 | 0.33 | -15.13 | 0.14 |
|  | Urinary tract disease | Low level | 81.57*** | 0.09 | -2.99 | 0.05 |
|  |  | Medium level | 156.13*** | 0.12 | -3.12 | 0.07 |
|  |  | High level | 156.15*** | 0.21 | -30.67** | 0.16 |
| Urology | Prostata disease | Low level | 56.42*** | 0.11 | 1.57 | 0.03 |
|  |  | Medium level | 55.08*** | 0.14 | 0.73 | 0.03 |
|  |  | High level | 103.65*** | 0.20 | 7.77 | 0.05 |
|  | Urinary tract disease | Low level | 131.38*** | 0.12 | 1.58 | 0.03 |
|  |  | Medium level | 155.4*** | 0.12 | 6.42 | 0.03 |
|  |  | High level | 130.48*** | 0.16 | 8.46 | 0.05 |
| Surgery | Injury | Low level | 17.21 | 0.10 | 3.01 | 0.08 |
|  |  | Medium level | 10.25 | 0.15 | -20.15 | 0.12 |
|  |  | High level | 88.72*** | 0.21 | 9.33 | 0.17 |
|  | Skin disease | Low level | 15.26 | 0.13 | 13.96 | 0.12 |
|  |  | Medium level | 5.21 | 0.24 | -22.24 | 0.20 |
|  |  | High level | 84.56** | 0.26 | 47.81 | 0.24 |
| Dermatology | Skin disease | Low level | 30.62*** | 0.09 | -1.08 | 0.04 |
|  |  | Medium level | 43.16*** | 0.11 | -2.01 | 0.05 |
|  |  | High level | 43.20** | 0.17 | 2.06 | 0.07 |
|  | Bedsore/decubitus | Low level | 60.84*** | 0.13 | -5.45 | 0.06 |
|  |  | Medium level | 93.37*** | 0.15 | -7.15 | 0.08 |
|  |  | High level | 213.96*** | 0.19 | -1.10 | 0.10 |
| Otolaryngology | Disease of the ear | Low level | 35.85*** | 0.09 | -3.61 | 0.03 |
|  |  | Medium level | 44.90*** | 0.12 | 0.95 | 0.04 |
|  |  | High level | 101.64*** | 0.16 | 5.80 | 0.06 |
| Nephrology | Renal failure | Low level | -13.10 | 0.08 | 29.94*** | 0.06 |
|  |  | Medium level | 17.27 | 0.10 | 38.48*** | 0.10 |
|  |  | High level | 61.58** | 0.19 | 51.06*** | 0.16 |
| Pneumology | Respiratory disease | Low level | 29.13*** | 0.07 | 7.65 | 0.05 |
|  |  | Medium level | 63.87*** | 0.11 | -1.12 | 0.08 |
|  |  | High level | 156.25*** | 0.24 | 10.13 | 0.23 |
| Psychiatry / Neurology | Parkinson´s diseases | Low level | -11.79 | 0.10 | 5.65** | 0.03 |
|  |  | Medium level | -14.29 | 0.13 | 12.65*** | 0.03 |
|  |  | High level | -27.12 | 0.17 | 18.58*** | 0.04 |
|  | Delusional/ personality disorders | Low level | -31.76*** | 0.14 | 0.29 | 0.04 |
|  |  | Medium level | -42.78*** | 0.18 | 10.08** | 0.04 |
|  |  | High level | -61.53*** | 0.23 | 12.83** | 0.05 |
|  | Dementia-related disease | Low level | -38.38*** | 0.06 | 9.87*** | 0.02 |
|  |  | Medium level | -54.6*** | 0.07 | 14.21*** | 0.02 |
|  |  | High level | -58.43*** | 0.09 | 21.89*** | 0.03 |
|  | Palsy/paresis | Low level | 2.46 | 0.14 | 23.47*** | 0.05 |
|  |  | Medium level | -20.74 | 0.14 | 29.03*** | 0.05 |
|  |  | High level | -38.21*** | 0.18 | 28.17*** | 0.06 |
|  | Depression | Low level | -15.91*** | 0.06 | 6.60*** | 0.02 |
|  |  | Medium level | -34.14*** | 0.08 | 13.16*** | 0.03 |
|  |  | High level | -42.79*** | 0.12 | 14.65*** | 0.03 |
|  | Neurosis | Low level | -12.56 | 0.08 | 3.18 | 0.03 |
|  |  | Medium level | -36.07*** | 0.11 | 8.26** | 0.03 |
|  |  | High level | -46.87*** | 0.18 | 14.32*** | 0.05 |
|  | Mono- and polyneuropathy | Low level | 3.78 | 0.08 | 9.31*** | 0.03 |
|  |  | Medium level | -4.98 | 0.10 | 12.51*** | 0.04 |
|  |  | High level | -15.88 | 0.17 | 18.20*** | 0.06 |
|  | Cerebrovascular disease | Low level | -6.53 | 0.07 | 13.03*** | 0.03 |
|  |  | Medium level | -31.22*** | 0.09 | 13.58*** | 0.03 |
|  |  | High level | -36.18*** | 0.11 | 23.78*** | 0.04 |
|  | Disorders due to psychoactive substance use | Low level | -23.37** | 0.13 | 13.44** | 0.05 |
|  |  | Medium level | -20.04 | 0.19 | 12.35 | 0.07 |
|  |  | High level | -70.80*** | 0.29 | 9.91 | 0.08 |

*Notes:* Alpha level: *** ≤ 0.01; ** ≤ 0.05; * ≤ 0.1; shown are percentage changes in risk/intensity of care; SE = standard error
Low level = i.e. German „Pflegestufe 1“, medium level =„Pflegestufe 2“, high level = „Pflegestufe 3“ and hardship cases; reference group = elderly not in need of long-term care

Further covariates in the model: gender, age, mortality, general practitioner visits, type of residential location and morbidity

^‡^based on logistic regression analysis
