# Supplementary material for: Differences in medical specialist utilization among older people in need of long-term care – results from German health claims data
Source: Int J Equity Health. 2020 Feb 7;19:22. doi: 10.1186/s12939-020-1130-z (PMC7006141; doi:10.1186/s12939-020-1130-z)
Supplement: Supplementary file 4 — Additional file 4. Association between GP visits and medical specialist visits. [file 12939_2020_1130_MOESM4_ESM.docx]

Additional File 4: Association between GP visits and medical specialist visits

| **Medical specialty** | **Disease category** | **Effect of GP visits on the** | | | |  |
| --- | --- | --- | --- | --- | --- | --- |
|  |  | **having no visit** | | **intensity of care** | | **McFadden pseudo R²** |
|  |  | **% increase** | **SE** | **% increase** | **SE** |  |
| Internal medicine | Renal failure | -0.65 | 0.01  0.01  0.01  0.01  0.01  0.01  0.01  0.01  0.01  0.01  0.01  0.02  0.01  0.00  0.02  0.01  0.01  0.01  0.02  0.01  0.01  0.01  0.01  0.02  0.02  0.01  0.01  0.01  0.02  0.01  0.02  0.01  0.01  0.01  0.02  0.01  0.02  0.02  0.01  0.01  0.01  0.01  0.02  0.01 | -2.96*** | 0.01  0.00  0.00  0.01  0.01  0.01  0.00  0.00  0.00  0.00  0.01  0.01  0.00  0.00  0.02  0.01  0.01  0.01  0.00  0.00  0.00  0.01  0.00  0.00  0.01  0.00  0.00  0.01  0.02  0.00  0.01  0.00  0.01  0.01  0.00  0.01  0.00  0.00  0.00  0.00  0.00  0.00  0.01  0.01 | 0.068 |
|  |  |  |  |  |  |  |
|  |  |  |  |  |  |  |
|  | Respiratory disease | -4.69*** | 0.01 | -2.37*** | 0.00 | 0.056 |
|  |  |  |  |  |  |  |
|  |  |  |  |  |  |  |
|  | Heart disease | -3.82*** | 0.01 | -3.37*** | 0.00 | 0.066 |
|  |  |  |  |  |  |  |
|  |  |  |  |  |  |  |
|  | Mono- and polyneuropathy | -1.81** | 0.01 | -3.36*** | 0.01 | 0.076 |
|  |  |  |  |  |  |  |
|  |  |  |  |  |  |  |
|  | Nutrition-related disease | -4.21*** | 0.01 | -3.09*** | 0.01 | 0.083 |
|  |  |  |  |  |  |  |
|  |  |  |  |  |  |  |
|  | Cerebrovascular disease | -3.14** | 0.01 | -3.91*** | 0.01 | 0.101 |
|  |  |  |  |  |  |  |
|  |  |  |  |  |  |  |
|  | Coronary disease | -3.77*** | 0.01 | -3.28*** | 0.00 | 0.079 |
|  |  |  |  |  |  |  |
|  |  |  |  |  |  |  |
|  | Intestinal disease | -4.81*** | 0.01 | -2.48*** | 0.00 | 0.074 |
|  |  |  |  |  |  |  |
|  |  |  |  |  |  |  |
|  | Metabolic disorders | -3.65*** | 0.01 | -3.51*** | 0.00 | 0.083 |
|  |  |  |  |  |  |  |
|  |  |  |  |  |  |  |
|  | Diabetes mellitus | -1.92** | 0.01 | -3.96*** | 0.00 | 0.083 |
|  |  |  |  |  |  |  |
|  |  |  |  |  |  |  |
|  | Thyroid disorders | -2.90*** | 0.01 | -4.19*** | 0.01 | 0.077 |
|  |  |  |  |  |  |  |
|  |  |  |  |  |  |  |
|  | Parkinson´s disease | -2.83* | 0.02 | -1.18 | 0.01 | 0.110 |
|  |  |  |  |  |  |  |
|  |  |  |  |  |  |  |
|  | Arthropathy | -5.19*** | 0.01 | -2.60*** | 0.00 | 0.080 |
|  |  |  |  |  |  |  |
|  |  |  |  |  |  |  |
|  | Hypertension | -3.75*** | 0.00 | -4.16*** | 0.00 | 0.085 |
|  |  |  |  |  |  |  |
|  |  |  |  |  |  |  |
|  | Motor impairment^‡^ | - | - | - | - | 0.171 |
|  |  |  |  |  |  |  |
|  |  |  |  |  |  |  |
|  | Palsy/paresis | -4.08** | 0.02 | -1.37 | 0.02 | 0.113 |
|  |  |  |  |  |  |  |
|  |  |  |  |  |  |  |
| Cardiology | Heart disease | -7.56*** | 0.01 | -1.01* | 0.01 | 0.046 |
|  |  |  |  |  |  |  |
|  |  |  |  |  |  |  |
|  | Coronary disease | -8.95*** | 0.01 | -1.58** | 0.01 | 0.109 |
|  |  |  |  |  |  |  |
|  |  |  |  |  |  |  |
|  | Hypertension | -7.95*** | 0.01 | -1.49*** | 0.01 | 0.113 |
|  |  |  |  |  |  |  |
|  |  |  |  |  |  |  |
| Ophthalmology | Diseases of the eye | -8.70*** | 0.02 | 0.30 | 0.00 | 0.023 |
|  |  |  |  |  |  |  |
|  |  |  |  |  |  |  |
| Orthopedics | Osteopathy and chondropathy | -3.69** | 0.01 | -1.36*** | 0.00 | 0.076 |
|  |  |  |  |  |  |  |
|  |  |  |  |  |  |  |
|  | Arthropathy | -4.08*** | 0.01 | -0.93*** | 0.00 | 0.064 |
|  |  |  |  |  |  |  |
|  |  |  |  |  |  |  |
|  | Injury | -4.96*** | 0.01 | -1.23** | 0.01 | 0.100 |
|  |  |  |  |  |  |  |
|  |  |  |  |  |  |  |
|  | Spinal disease | -4.39*** | 0.01 | -0.84*** | 0.00 | 0.065 |
|  |  |  |  |  |  |  |
|  |  |  |  |  |  |  |
|  | Motor impairment^‡^ | - | - | - | - | 0.211 |
|  |  |  |  |  |  |  |
|  |  |  |  |  |  |  |
| Gynecology | Diseases of the female genital tract | 1.83 | 0.02 | 0.73* | 0.00 | 0.028 |
|  |  |  |  |  |  |  |
|  |  |  |  |  |  |  |
|  | Urinary tract disease | -1.87 | 0.02 | -0.01 | 0.01 | 0.192 |
|  |  |  |  |  |  |  |
|  |  |  |  |  |  |  |
| Urology | Prostata disease | 1.89* | 0.01 | 0.34 | 0.00 | 0.048 |
|  |  |  |  |  |  |  |
|  |  |  |  |  |  |  |
|  | Urinary tract disease | 0.90 | 0.01 | 0.61** | 0.00 | 0.068 |
|  |  |  |  |  |  |  |
|  |  |  |  |  |  |  |
| Surgery | Injury | -0.98 | 0.01 | 0.62 | 0.01 | 0.031 |
|  |  |  |  |  |  |  |
|  |  |  |  |  |  |  |
|  | Skin disease | -3.04 | 0.02 | 1.98 | 0.02 | 0.047 |
|  |  |  |  |  |  |  |
|  |  |  |  |  |  |  |
| Dermatology | Skin disease | -0.71 | 0.01 | 0.48 | 0.00 | 0.026 |
|  |  |  |  |  |  |  |
|  |  |  |  |  |  |  |
|  | Bedsore/decubitus | 0.74 | 0.02 | -0.13 | 0.01 | 0.101 |
|  |  |  |  |  |  |  |
|  |  |  |  |  |  |  |
| Otolaryngology | Disease of the ear | -7.04*** | 0.01 | 0.08 | 0.00 | 0.016 |
|  |  |  |  |  |  |  |
|  |  |  |  |  |  |  |
| Nephrology | Renal failure | 4.31*** | 0.01 | -1.99** | 0.01 | 0.056 |
|  |  |  |  |  |  |  |
|  |  |  |  |  |  |  |
| Pneumology | Respiratory disease | -2.63** | 0.01 | -1.29** | 0.01 | 0.034 |
|  |  |  |  |  |  |  |
|  |  |  |  |  |  |  |
| Psychiatry/  Neurology | Parkinson´s diseases | -1.90 | 0.02 | -1.10** | 0.00 | 0.059 |
|  |  |  |  |  |  |  |
|  |  |  |  |  |  |  |
|  | Delusional/ personality disorders | -0.22 | 0.01 | -0.42 | 0.01 | 0.073 |
|  |  |  |  |  |  |  |
|  |  |  |  |  |  |  |
|  | Dementia-related disease | -0.24 | 0.02 | -1.10** | 0.00 | 0.088 |
|  |  |  |  |  |  |  |
|  |  |  |  |  |  |  |
|  | Palsy/paresis | -0.52 | 0.02 | -0.38 | 0.00 | 0.111 |
|  |  |  |  |  |  |  |
|  |  |  |  |  |  |  |
|  | Depression | -1.19 | 0.01 | -0.94*** | 0.00 | 0.086 |
|  |  |  |  |  |  |  |
|  |  |  |  |  |  |  |
|  | Neurosis | -2.41** | 0.01 | -1.31*** | 0.00 | 0.105 |
|  |  |  |  |  |  |  |
|  |  |  |  |  |  |  |
|  | Mono- and polyneuropathy | -1.71 | 0.01 | -1.00** | 0.00 | 0.108 |
|  |  |  |  |  |  |  |
|  |  |  |  |  |  |  |
|  | Cerebrovascular disease | -2.45** | 0.01 | 0.17 | 0.00 | 0.130 |
|  |  |  |  |  |  |  |
|  |  |  |  |  |  |  |
|  | Disorders due to psychoactive substance use | -2.85* | 0.02 | -0.87* | 0.01 | 0.164 |
|  |  |  |  |  |  |  |
|  |  |  |  |  |  |  |

*Notes:* Alpha level: *** ≤ 0.01; ** ≤ 0.05; * ≤ 0.1; shown are percentage increases in risk/intensity of care. SE = standard error

Further covariates in the model: long-term care setting and level of long-term care need, gender, age, mortality, general practitioner visits, type of residential location and morbidity

^‡^based on logistic regression analysis
